# Supplementary material for: Managerial capacity among district health managers and its association with district performance: A comparative descriptive study of six districts in the Eastern Region of Ghana
Source: PLoS One. 2020 Jan 22;15(1):e0227974. doi: 10.1371/journal.pone.0227974 (PMC6975551; doi:10.1371/journal.pone.0227974)
Supplement: S1 File — (DOCX) [file pone.0227974.s001.docx]

**Questionnaire**

**District Health Managers’ Self-Assessed Management Capacity**

***Dear Respondent***,

We greatly appreciate that you are taking the time to complete this questionnaire that is part of the PERFORM2Scale project “Improving Health Workforce Performance to Achieve Universal Health Coverage”. The project is carried out in collaboration with the *School of Public Health, University of Ghana.*PERFORM2Scale aims at strengthening District Health Management to improve the workforce performance through a Management Strengthening Intervention using an Action Research approach (*Plan-Act-Study-Reflect*). This questionnaire investigates health managers’ perceived management capacity at District level at baseline, before implementation of the Management Strengthening Intervention, and will be repeated at the end of the project in 2020.

The survey is divided into 7 parts, and will take maximum 30-45 minutes to complete.

*1) Socio-demographic Information*

*2) Previous Management Experience/Training*

*3) Functioning Support Systems*

*4) General Management Skills and Competencies*

*5) Specific Health System Management Skills and Competencies*

*6) Overall Management Competency*

*7) Being part of the District Health Management Team*

Your responses are voluntary and all information obtained via this study will remain **confidential**. In any written reports or publications, all responses will be compiled together and analyzed and reported as a group. You are free to withdraw your participation at any time.

**We greatly appreciate your valuable contribution. Thank you!**

***To be filled in by CRT***

| ID Questionnaire: |  |
| --- | --- |
| District : |  |
| Date (DD/MM/YY): |  |
| Informed consent | Yes :___ No:____ |

| **Part 1. Socio-demographic Information** |
| --- |

| 1. Name of District |  | | | | | |
| --- | --- | --- | --- | --- | --- | --- |
| 1. Sex | ☐ | Male *(1)* | ☐ | Female *(2)* | | |
| 1. How old are you? | _____ years | | | | | |
| 1. Current position | ☐ | Accountant *(1)* | | | | |
|  | ☐ | Administrator *(2)* | | | | |
|  | ☐ | Deputy Director of Nursing Services (3) | | | | |
|  | ☐ | Disease Control Officer (4) | | | | |
|  | ☐ | District Director of Health Services *(5)* | | | | |
|  | ☐ | Health Information Officer *(6)* | | | | |
|  | ☐ | Health Promotion Officer *(7)* | | | | |
|  | ☐ | Human Resource Officer *(8)* | | | | |
|  | ☐ | Nutrition Officer *(9)* | | | | |
|  | ☐ | Pharmacist *(10)* | | | | |
|  | ☐ | Principal Nursing Officer *(11)* | | | | |
|  | ☐ | Public Health Nurse *(12)* | | | | |
|  | ☐ | Other, *please specify (13)* | | | | |
| 1. What is your employment status? | ☐ | Full time *(1)* | | | ☐ | Part-time *(2)* |
| 1. Do you have a supervisory role? | ☐ | Yes *(1)* | | | ☐ | No *(2)* |
| 1. What is your educational background? *(multiple answers allowed)* | ☐ | Public Health *(1)* | | | ☐ | Medical Doctor *(2)* |
|  | ☐ | Nursing *(3)* | | | ☐ | Midwifery *(4)* |
|  | ☐ | Accounting /Finance *(5)* | | | ☐ | HR Management *(6)* |
|  | ☐ | Nutrition *(7)* | | | ☐ | Other, *please specify*  *(8)* |
| 1. What is your highest qualification? | ☐ | Certificate *(1)* | | | ☐ | Diploma *(2)* |
|  | ☐ | 1st degree (Bachelor) *(3)* | | | ☐ | 2nd degree (Master) *(4)* |
|  | ☐ | PhD *(5)* | | | ☐ | Other, *please specify (6)* |

| **Part 2. Previous Management Experience and Training** |
| --- |

| 1. How long have you been in your current position | ____years | | | | | | | |
| --- | --- | --- | --- | --- | --- | --- | --- | --- |
| 1. How did you get into your current position? | ☐ | | I applied for my position *(1)* | | | | | |
|  | ☐ | | I was assigned to my position without applying *(2)* | | | | | |
| 1. Prior to your current position in the DHMT, did you have any professional experience of being in a management position? | ☐ | No experience *(1)* | | | ☐ | | Less than 1 year of experience *(2)* | |
|  | ☐ | 1-5 years of experience *(3)* | | | ☐ | | 5+ years of experience *(4)* | |
| 1. Prior to your current position, did you work in another DHMT? | ☐ | Yes *(1)* | | ☐ | No, *please go to question 14*  *(2)* | | | |
| 1. *If yes to question 12*, please specify where and which position you held | District: | | | | | | | |
|  | Region: | | | | | | | |
|  | Position: | | | | | | | |
| 1. Have you received any formal training in management and/or leadership *( i.e.* *certificate, diploma or degree)* | ☐ | Yes, *please specify the type of training (1)*  ………………………………….. | | | | | | |
|  | ☐ | No *(2)* | | | | | | |
| 1. Have you received any informal training aimed at strengthening your management and/or leadership skills within the last 12 months? (*i.e.* *mentoring, in-service training, non-certified programs)* | ☐ | Yes *(1)* | | | | | | |
|  | ☐ | No *(please go to question 18) (2)* | | | | | | |
| 1. *If yes to question 15*, how many days of training did you undertake over the past 12 months | ☐ | 1 day (or less) *(1)* | | | | ☐ | | 2-5 days *(2)* |
|  | ☐ | 6-10 days *(3)* | | | | ☐ | | More than 10 days *(4)* |
| 1. Who initiated/required the training? *(multiple answers allowed)* | ☐ | Ministry of Health*(1)* | | | | ☐ | | Ghana Health Service *(2)* |
|  | ☐ | NGOs *(3)* | | | | ☐ | | Academic Institutions *(4)* |
|  | ☐ | Other, *please specify (5)* | | | | | | |

| **Part 3: Functional Support Systems** |
| --- |

| 1. Have you been provided with a job description? | ☐ | Yes *(1)* | ☐ | No, *please go to question 21 (2)* | | |
| --- | --- | --- | --- | --- | --- | --- |
| 1. Is your job description up-to date and accurate in terms of your roles and responsibilities? | ☐ | Yes *(1)* | ☐ | No *(2)* | | |
| 1. Do you take on any additional roles and responsibilities beside what is stated in your job description? | ☐ | No, not at all *(1)* | ☐ | Yes, to a moderate extent *(2)* | ☐ | Yes, to a large extent *(3)* |

| **Is the following available to help you in carrying out your role and responsibilities?** *If you do not have a need for the following items please tick ☑ NA* | | | | | |
| --- | --- | --- | --- | --- | --- |
|  | ***Not at all*** *(1)* | ***To a small extent*** *(2)* | ***To a moderate extent*** *(3)* | ***To a large extent*** *(4)* | ***N/A*** *(5)* |
| 1. Access to relevant national and/or regional guidelines within your work area | ☐ | ☐ | ☐ | ☐ | ☐ |
| 1. Regular team meetings | ☐ | ☐ | ☐ | ☐ | ☐ |
| 1. Records of team meetings available (i.e. minutes) | ☐ | ☐ | ☐ | ☐ | ☐ |
| 1. Adequate funds to carry out planned activities | ☐ | ☐ | ☐ | ☐ | ☐ |
| 1. Adequate logistics and infrastructure to carry out planned activities | ☐ | ☐ | ☐ | ☐ | ☐ |
| 1. Supportive supervision, feedback and mentoring from your supervisor | ☐ | ☐ | ☐ | ☐ | ☐ |
| **In case you need help with completing an assignment, is there a system in place to support you within the following areas?** | | | | | |
| 1. Planning and budgeting | ☐ | ☐ | ☐ | ☐ | ☐ |
| 1. Procurement of drugs and other commodities | ☐ | ☐ | ☐ | ☐ | ☐ |
| 1. Data management | ☐ | ☐ | ☐ | ☐ | ☐ |
| 1. HR management | ☐ | ☐ | ☐ | ☐ | ☐ |
| 1. Community-level structures or groups that enable community involvement | ☐ | ☐ | ☐ | ☐ | ☐ |

| **Part 4. General Management Skills and Competencies** |
| --- |

*Please indicate your management competencies by ticking ☑ the appropriate box, not leaving any blank.*

|  | ***Strongly disagree*** *(1)* | | ***Disagree*** *(2)* | ***Neutral*** *(3)* | ***Agree*** *(4)* | ***Strongly Agree*** *(5)* | | ***N/A (****6)* |
| --- | --- | --- | --- | --- | --- | --- | --- | --- |
| *Interpersonal skills* | | | | | | | | |
| 1. I have a clear understanding of the roles and responsibilities of members within the DHMT | ☐ | | ☐ | ☐ | ☐ | ☐ | | ☐ |
| 1. I ensure that staff under my supervision feel their contributions are valued and appreciated | ☐ | | ☐ | ☐ | ☐ | ☐ | | ☐ |
| *Leadership skills* | | | | | | | | |
| 1. I set clear and measurable targets for myself and staff under my supervision | ☐ | | ☐ | ☐ | ☐ | ☐ | | ☐ |
| 1. I encourage feedback on my performance and reflect and act on received suggestions | ☐ | | ☐ | ☐ | ☐ | ☐ | | ☐ |
| 1. I actively seek opportunities to learn and develop | ☐ | | ☐ | ☐ | ☐ | ☐ | | ☐ |
| 1. I am confident in my abilities to direct and motivate people I work with | ☐ | | ☐ | ☐ | ☐ | ☐ | | ☐ |
| 1. I delegate work appropriately to staff, and provide them with the necessary support to meet their objectives | ☐ | | ☐ | ☐ | ☐ | ☐ | | ☐ |
| *Conflict Handling skills* | | | | | | | | |
| 1. I feel confident in managing and resolving conflicts when they arise | ☐ | ☐ | | ☐ | ☐ | ☐ | | ☐ |
| *Time Planning Skills* | | | | | | | | |
| 1. I plan my workload by setting up daily/weekly/monthly to-do-lists | ☐ | ☐ | | ☐ | ☐ | | ☐ | ☐ |
| 1. I always deliver my commitments to high standards (on time, complete) | ☐ | ☐ | | ☐ | ☐ | | ☐ | ☐ |
| 1. When unplanned/adhoc events arise from the regional/national level, I am able to fit it into my plans | ☐ | ☐ | | ☐ | ☐ | | ☐ | ☐ |

| **Part 5. Specific Health System Management Skills and Competencies** |
| --- |

**1.** **Oversight & Coordination**

| **Please indicate your level of involvement in the following activities**  *If you are not involved in the following activities please tick ☑ “Not at all”* | | | | |
| --- | --- | --- | --- | --- |
|  | ***Not at all*** *(1)* | ***To a small extent*** *(2)* | ***To a moderate extent*** *(3)* | ***To a large extent*** *(4)* |
| 1. Identifying health service delivery gaps within your work area | ☐ | ☐ | ☐ | ☐ |
| 1. Planning activities to address gaps within your work area | ☐ | ☐ | ☐ | ☐ |
| 1. Mobilizing resources to address gaps within your work area | ☐ | ☐ | ☐ | ☐ |
| 1. Supervising health facilities at sub-district level | ☐ | ☐ | ☐ | ☐ |
| 1. Monitoring stakeholder activities within the district | ☐ | ☐ | ☐ | ☐ |
| 1. Providing feedback to stakeholders, health facilities and communities | ☐ | ☐ | ☐ | ☐ |
| 1. Attending coordinating meetings and workshops | ☐ | ☐ | ☐ | ☐ |
| **If you indicated a level of involvement in the above statements, please assess your management competencies below by selecting whether you agree or disagree with the listed statements.** *If you do not have authority or responsibilities within certain areas in the following please tick ☑ NA* | | | | |

|  | ***Strongly disagree****(1)* | ***Disagree*** *(2)* | ***Neutral*** *(3)* | ***Agree*** *(4)* | ***Strongly Agree*** *(5)* | ***N/A*** *(6)* |
| --- | --- | --- | --- | --- | --- | --- |
| *Situation Analysis* | | | | | | |
| 1. I have a good understanding of national guidelines, strategies and policies relating to my work area | ☐ | ☐ | ☐ | ☐ | ☐ | ☐ |
| 1. I am well informed about health-related activities and strategies of a wide number of stakeholders within my work area | ☐ | ☐ | ☐ | ☐ | ☐ | ☐ |
| 1. I know how to collect and review data on health priorities within my work area from a range of sources | ☐ | ☐ | ☐ | ☐ | ☐ | ☐ |
| 1. I am confident in carrying out a situational analysis | ☐ | ☐ | ☐ | ☐ | ☐ | ☐ |
| *Problem Analysis & Problem Statement* | | | | | |  |
| 1. I am confident in identifying areas in need of improvement within my work area | ☐ | ☐ | ☐ | ☐ | ☐ | ☐ |
| 1. I am able to identify the root causes of problems | ☐ | ☐ | ☐ | ☐ | ☐ | ☐ |
| 1. I am able to prioritize which of the identified areas are most important to address | ☐ | ☐ | ☐ | ☐ | ☐ | ☐ |
| 1. When analysing problems I take the different needs of males and females into account | ☐ | ☐ | ☐ | ☐ | ☐ | ☐ |
| 1. I am able to state a clear and specific problem statement | ☐ | ☐ | ☐ | ☐ | ☐ | ☐ |
| 1. I am confident in my abilities to develop effective strategies to address an identified problem | ☐ | ☐ | ☐ | ☐ | ☐ | ☐ |
| *Planning* | | | | | | |
| 1. I am able to translate the district plan into action plans | ☐ | ☐ | ☐ | ☐ | ☐ | ☐ |
| 1. I am able to translate action plans into weekly/monthly activities | ☐ | ☐ | ☐ | ☐ | ☐ | ☐ |
| 1. When planning, I engage with and involve stakeholders in implementing health-related activities in the district | ☐ | ☐ | ☐ | ☐ | ☐ | ☐ |
| 1. When planning, I assess benefits and risks of available options | ☐ | ☐ | ☐ | ☐ | ☐ | ☐ |
| 1. When planning, I always pay special attention to the health needs of vulnerable population groups | ☐ | ☐ | ☐ | ☐ | ☐ | ☐ |
| 1. I consider how a strategy affect males and females differently | ☐ | ☐ | ☐ | ☐ | ☐ | ☐ |
| 1. I develop indicators to track the effects that strategies have | ☐ | ☐ | ☐ | ☐ | ☐ | ☐ |
| *Implementation and Monitoring* | | | | | | |
| 1. I try out new and innovative strategies to address identified priorities | ☐ | ☐ | ☐ | ☐ | ☐ | ☐ |
| 1. I use information from a range of sources to monitor progress of agreed plans | ☐ | ☐ | ☐ | ☐ | ☐ | ☐ |
| 1. I am able to adapt strategies and add new strategies if they don’t achieve the intended results | ☐ | ☐ | ☐ | ☐ | ☐ | ☐ |

| *Reporting* | | | | | | |
| --- | --- | --- | --- | --- | --- | --- |
| 1. I feel confident in using data to produce and deliver credible and understandable reports | ☐ | ☐ | ☐ | ☐ | ☐ | ☐ |
| 1. I ensure that reporting is done to a high standard (complete, on time) | ☐ | ☐ | ☐ | ☐ | ☐ | ☐ |

**2.** **Human Resource Management**

| **Please indicate your level of involvement in the following activities**  *If you are not involved in the following activities please tick ☑ “Not at all”* | | | | |  |
| --- | --- | --- | --- | --- | --- |
|  | ***Not at all*** *(1)* | ***To a small extent*** *(2)* | ***To a moderate extent*** *(3)* | ***To a large extent*** *(4)* |  |
| 1. Identifying human resource needs (management and clinical) | ☐ | ☐ | ☐ | ☐ |  |
| 1. Posting and reposting human resources within the district | ☐ | ☐ | ☐ | ☐ |  |
| 1. Monitoring of personnel performance | ☐ | ☐ | ☐ | ☐ |  |
| **If you indicated a level of involvement in the above statements, please assess your management competencies below by selecting whether you agree or disagree with the listed statements.** *If you do not have authority or responsibilities within certain areas in the following please tick ☑ NA* | | | | | |

|  | ***Strongly disagree****(1)* | ***Disagree*** *(2)* | ***Neutral*** *(3)* | ***Agree*** *(4)* | ***Strongly Agree*** *(5)* | ***N/A*** *(6)* |
| --- | --- | --- | --- | --- | --- | --- |
| 1. I am confident in my ability to identify human resource needs in my district | ☐ | ☐ | ☐ | ☐ | ☐ | ☐ |
| 1. I am able to effectively lobby for new staff to the District Director/MoH/Local Government/Partners | ☐ | ☐ | ☐ | ☐ | ☐ | ☐ |
| 1. I am confident in my abilities to deploy staff in response to changing work requirements | ☐ | ☐ | ☐ | ☐ | ☐ | ☐ |
| 1. I avoid staff at facility level getting fatigued by making sure they all have regular time off | ☐ | ☐ | ☐ | ☐ | ☐ | ☐ |
| 1. I ensure that the health workforce have signed contracts/appointment letters | ☐ | ☐ | ☐ | ☐ | ☐ | ☐ |
| 1. I ensure that the health workforce have written job descriptions | ☐ | ☐ | ☐ | ☐ | ☐ | ☐ |
| 1. I ensure that the health workforce have clearly assigned supervisors | ☐ | ☐ | ☐ | ☐ | ☐ | ☐ |
| 1. I ensure that annual appraisal talks are carried out at facility level | ☐ | ☐ | ☐ | ☐ | ☐ | ☐ |

| 1. I ensure that the staff I supervise are supervised in a supportive way every quarter | ☐ | ☐ | ☐ | ☐ | ☐ | ☐ |
| --- | --- | --- | --- | --- | --- | --- |
| 1. I make sure all staff receive timely and specific feedback from supervision visits | ☐ | ☐ | ☐ | ☐ | ☐ | ☐ |
| 1. I allocate resources to further training/in-service training of the health workforce | ☐ | ☐ | ☐ | ☐ | ☐ | ☐ |

**3. Resource Management**

| **Please indicate your level of involvement in the following activities**  *If you are not involved in the following activities please tick ☑ “Not at all”* | | | | |
| --- | --- | --- | --- | --- |
|  | ***Not at all*** *(1)* | ***To a small extent*** *(2)* | ***To a moderate extent*** *(3)* | ***To a large extent*** *(4)* |
| 1. Managing regular supply of drugs at facilities (i.e. monitoring stocks, procurement distribution to facilities) | ☐ | ☐ | ☐ | ☐ |
| 1. Managing regular supply of medical and nonmedical supplies at facilities (i.e. monitoring stocks, procurement, maintenance, distribution to facilities) | ☐ | ☐ | ☐ | ☐ |
| **If you indicated a level of involvement in the above statements, please assess your management competencies below by selecting whether you agree or disagree with the listed statements.** *If you do not have authority or responsibilities within certain areas in the following please tick ☑ NA* | | | | |

|  | ***Strongly disagree****(1)* | ***Disagree*** *(2)* | ***Neutral*** *(3)* | ***Agree*** *(4)* | ***Strongly Agree*** *(5)* | ***N/A*** *(6)* |
| --- | --- | --- | --- | --- | --- | --- |
| 1. I closely follow up on the availability of medical and/or non-medical supplies to avoid stock outs | ☐ | ☐ | ☐ | ☐ | ☐ | ☐ |
| 1. I closely follow up on the availability of drugs to avoid stock outs | ☐ | ☐ | ☐ | ☐ | ☐ | ☐ |
| 1. I ensure key equipment is available at facilities | ☐ | ☐ | ☐ | ☐ | ☐ | ☐ |
| 1. I ensure that preventive maintenance of equipment is taking place regularly | ☐ | ☐ | ☐ | ☐ | ☐ | ☐ |

**4. Financial Management**

| **Please indicate your level of involvement in the following activities within your work area**  *If you are not involved in the following activities please tick ☑ “Not at all”* | | | | |
| --- | --- | --- | --- | --- |
|  | ***Not at all (1)*** | ***To a small extent (2)*** | ***To a moderate extent (3)*** | ***To a large extent (4)*** |
| 1. Developing and managing budgets | ☐ | ☐ | ☐ | ☐ |
| 1. Mobilizing financial resources | ☐ | ☐ | ☐ | ☐ |
| 1. Financial reporting | ☐ | ☐ | ☐ | ☐ |
| **If you indicated a level of involvement in the above statements, please assess your management competencies below by selecting whether you agree or disagree with the listed statements.** *If you do not have authority or responsibilities within certain areas in the following please tick ☑ NA* | | | | |

|  | ***Strongly disagree****(1)* | ***Disagree*** *(2)* | ***Neutral*** *(3)* | ***Agree*** *(4)* | ***Strongly Agree*** *(5)* | ***N/A*** *(6)* |
| --- | --- | --- | --- | --- | --- | --- |
| 1. I am confident in my own abilities to prepare and manage budgets effectively | ☐ | ☐ | ☐ | ☐ | ☐ | ☐ |
| 1. I am confident in my own abilities to mobilize financial resources to tackle priority problems | ☐ | ☐ | ☐ | ☐ | ☐ | ☐ |
| 1. I ensure that financial reporting is done to a high standard (complete, on time) | ☐ | ☐ | ☐ | ☐ | ☐ | ☐ |

**5. Information Management**

| **Please indicate your level of involvement in the following activities within your work area**  *If you are not involved in the following activities please tick ☑ “Not at all”* | | | | |
| --- | --- | --- | --- | --- |
|  | ***Not at all*** *(1)* | ***To a small extent*** *(2)* | ***To a moderate extent*** *(3)* | ***To a large extent*** *(4)* |
| 1. Gathering, analysing and reporting information from facilities, stakeholders and communities to improve health services | ☐ | ☐ | ☐ | ☐ |
| **If you indicated a level of involvement in the above statements, please assess your management competencies below by selecting whether you agree or disagree with the listed statements.** *If you do not have authority or responsibilities within certain areas in the following please tick ☑ NA* | | | | |

|  | ***Strongly disagree****(1)* | ***Disagree*** *(2)* | ***Neutral*** *(3)* | ***Agree*** *(4)* | ***Strongly Agree*** *(5)* | ***N/A*** *(6)* |
| --- | --- | --- | --- | --- | --- | --- |
| 1. I ensure that relevant, complete and high quality data is collected and submitted on time | ☐ | ☐ | ☐ | ☐ | ☐ | ☐ |
| 1. I am able to manage collected data (i.e. safe storage, clean raw data, data analysis) | ☐ | ☐ | ☐ | ☐ | ☐ | ☐ |
| 1. I am able to use data to inform health planning in the district | ☐ | ☐ | ☐ | ☐ | ☐ | ☐ |

**6. Service Delivery and Community Involvement**

| **Please indicate your level of involvement in the following activities within your work area**  *If you are not involved in the following activities please tick ☑ “Not at all”* | | | | |
| --- | --- | --- | --- | --- |
|  | ***Not at all*** *(1)* | ***To a small extent*** *(2)* | ***To a moderate extent*** *(3)* | ***To a large extent*** *(4)* |
| 1. Involving the community in decisions about health activities and seeking their feedback on quality of health services | ☐ | ☐ | ☐ | ☐ |
| **If you indicated a level of involvement in the above statements, please assess your management competencies below by selecting whether you agree or disagree with the listed statements.** *If you do not have authority or responsibilities within certain areas in the following please tick ☑ NA* | | | | |

|  | ***Strongly disagree****(1)* | ***Disagree*** *(2)* | ***Neutral*** *(3)* | ***Agree*** *(4)* | ***Strongly Agree*** *(5)* | ***N/A*** *(6)* |
| --- | --- | --- | --- | --- | --- | --- |
| 1. I ensure that sub-district staff have access to and are familiar with guidelines and policies | ☐ | ☐ | ☐ | ☐ | ☐ | ☐ |
| 1. I ensure to involve and seek feedback from communities on the services that are provided | ☐ | ☐ | ☐ | ☐ | ☐ | ☐ |
| 1. I always take immediate action when I notice shortfalls in service delivery | ☐ | ☐ | ☐ | ☐ | ☐ | ☐ |

| **Part 6: Overall Management Performance** |
| --- |

| 1. Did you reach all your set annual objectives by the end of 2017? | ☐ | Yes | ☐ | No | | | |
| --- | --- | --- | --- | --- | --- | --- | --- |
| 1. Do you feel like your management and leadership competencies within certain areas could be improved? | ☐ | Yes | ☐ | No | | | |
| 1. *If yes to question 107*, please specify which competencies you would like to improve |  | | | | | | |
| 1. Overall, how would you rate your management and leadership skills and competencies? | ***Very poor*** *(1)* | | ***Poor*** *(2)* | | ***Fair*** *(3)* | ***Good*** *(4)* | ***Excellent*** *(5)* |
|  | ☐ | | ☐ | | ☐ | ☐ | ☐ |

| **Part 7: Being part of the District Health Management Team** |
| --- |

**Teamwork & Communication**

|  | ***Strongly disagree*** *(1)* | ***Disagree*** *(2)* | ***Neutral*** *(3)* | ***Agree*** *(4)* | ***Strongly agree*** *(5)* |
| --- | --- | --- | --- | --- | --- |
| 1. I really feel that I belong to a team | ☐ | ☐ | ☐ | ☐ | ☐ |
| 1. I look forward to being with the members of the DHMT each day | ☐ | ☐ | ☐ | ☐ | ☐ |
| 1. There is a lot of support and encouragement within the DHMT | ☐ | ☐ | ☐ | ☐ | ☐ |
| 1. It is very difficult to settle problems in the DHMT | ☐ | ☐ | ☐ | ☐ | ☐ |
| 1. The people I work with cooperate to get the job done | ☐ | ☐ | ☐ | ☐ | ☐ |
| 1. Team members keep their thoughts to themselves, rather than risk speaking out | ☐ | ☐ | ☐ | ☐ | ☐ |
| 1. I often work in groups as part of my job | ☐ | ☐ | ☐ | ☐ | ☐ |
| 1. There is frequent communication within the team | ☐ | ☐ | ☐ | ☐ | ☐ |
| 1. Team members communicate often through various channels (i.e spontaneous meetings, email, phone etc.) | ☐ | ☐ | ☐ | ☐ | ☐ |
| 1. Relevant information is shared openly by all team members | ☐ | ☐ | ☐ | ☐ | ☐ |
| 1. There has been conflict within our team regarding the openness of the information flow | ☐ | ☐ | ☐ | ☐ | ☐ |

**Job motivation, Job satisfaction & Organizational Commitment**

|  | ***Strongly disagree*** *(1)* | ***Disagree*** *(2)* | ***Neutral*** *(3)* | ***Agree*** *(4)* | ***Strongly agree*** *(5)* |
| --- | --- | --- | --- | --- | --- |
| 1. These days, I feel motivated to work as hard as I can | ☐ | ☐ | ☐ | ☐ | ☐ |
| 1. I only do this job so that I get paid at the end of the month | ☐ | ☐ | ☐ | ☐ | ☐ |
| 1. I do this job as it provides long term security for me | ☐ | ☐ | ☐ | ☐ | ☐ |
| 1. In general, I am satisfied with my job | ☐ | ☐ | ☐ | ☐ | ☐ |
| 1. I think there are many other jobs which are more interesting than mine | ☐ | ☐ | ☐ | ☐ | ☐ |
| 1. My current job fulfils the expectations I had before I started it | ☐ | ☐ | ☐ | ☐ | ☐ |
| 1. I would like to get another job because I am not satisfied | ☐ | ☐ | ☐ | ☐ | ☐ |
| 1. My current job is pleasant | ☐ | ☐ | ☐ | ☐ | ☐ |
| 1. I think my current job is interesting and fascinating | ☐ | ☐ | ☐ | ☐ | ☐ |
| 1. I am considering leaving this DHMT | ☐ | ☐ | ☐ | ☐ | ☐ |
| 1. Deciding to work for this DHMT was a definite mistake on my part | ☐ | ☐ | ☐ | ☐ | ☐ |
| 1. There is not too much to be gained by sticking with this DHMT indefinitely | ☐ | ☐ | ☐ | ☐ | ☐ |

**Thank you very much for your valuable contribution!**
