# Supplementary material for: Managerial capacity among district health managers and its association with district performance: A comparative descriptive study of six districts in the Eastern Region of Ghana
Source: PLoS One. 2020 Jan 22;15(1):e0227974. doi: 10.1371/journal.pone.0227974 (PMC6975551; doi:10.1371/journal.pone.0227974)
Supplement: S2 File — (PDF) [file pone.0227974.s002.pdf]

District Performance & Scorecard of Key PH Indicators.

Annual Performance Review 2017.

Introduction

- A **‘League of District Performance’** is illustrated for each indicator followed by a **‘Performance Gauge’** (PG). The PG as depicted below shows district performance for selected indicators based on a grading system **‘A-E’** and scored **‘5-1’** respectively. The table then ends with a ‘**League Table of the Overall Performance’** for the year 2017.

Methodology

- 17 core PH indicators are selected for assessment as a “League of District Performance”.
- Assessment is based on guidelines used by National to assess regions.
- Each indicator is assessed based on its own Performance Gauge
- Scores were assigned and aggregated to arrive at marks/positions for each quarter
- The range of scores spans
- 5 Excellent Deep Green
- 4 Very Good Light Green
- 3 Good Yellow
- 2 Satisfactory Orange
- 1 Unsatisfactory Red
- The total marks for the year is an aggregate for the 4Q’s.
- The average for each district is the total marks for the year divided by 4.
- The average number determines the position of the District.

Methodology cont;

Performance Gauge (PG)

Indicator				
Best range				Least
A	B	C	D	E
Excellent	Very Good	Good	Satisfactory	Unsatisfactory
Score				
5	4	3	2	1

DISTRICTS SCORECARD OF SELECTED INDICATORS, 1ST QUARTER 2017, ER.

District	OPD per capita	Percentage of Teenage Pregnancies among ANC registrants	Family Planning Acceptor rate	Percentage skilled deliveries	Measles-Rubella2 (MR-2) cov	Under 5 Malaria Case Fatality Rate	% Pregnant women tested HIV positive(PMTCT)	Penta 3 coverage	IPT	ANC	Authorisation Complteness	Authorisation Timelineness	IDSR Weekly Timelineness	IDSR Monthly Complteness	Non Polio AFP Rate	Data Entry Complteness	Data Entry Timelineness	G Total	Postion
[redacted]	5	3	4	5	5	5	4	5	1	5	5	5	2	5	1	2	3	65	1
[redacted]	5	1	4	4	1	5	4	5	2	5	5	5	3	5	1	3	4	62	2
[redacted]	5	1	3	4	2	5	4	5	2	4	5	5	3	5	1	3	3	60	3
[redacted]	2	1	5	1	5	5	5	5	2	5	5	5	3	5	1	2	1	58	4
[redacted]	5	2	5	3	3	5	4	5	2	3	5	5	2	5	1	1	2	58	4
[redacted]	5	4	4	4	4	5	5	5	3	2	1	1	1	5	1	3	4	57	6
[redacted]	3	1	3	1	1	5	5	5	5	2	5	5	4	5	1	2	3	56	7
[redacted]	1	5	3	1	5	5	2	5	2	1	5	1	4	5	1	3	3	52	8
[redacted]	2	1	5	1	5	5	1	4	2	2	5	1	3	5	1	4	5	52	8
[redacted]	2	1	4	1	2	5	5	5	3	1	5	1	3	5	1	3	4	51	10
[redacted]	2	3	3	1	3	5	1	5	3	1	5	1	3	5	1	3	3	48	11
[redacted]	2	1	4	1	2	5	4	5	1	1	5	1	3	5	1	4	3	48	11
[redacted]	5	2	4	3	2	5	2	2	1	1	5	1	4	5	1	2	2	47	13
[redacted]	1	1	2	1	4	5	5	5	1	1	1	1	4	5	1	3	5	46	14
[redacted]	3	1	1	5	4	5	1	2	2	5	1	1	3	5	1	2	4	46	14
[redacted]	1	1	3	1	2	5	3	5	1	3	5	1	1	5	1	2	5	45	16
[redacted]	2	2	5	1	1	5	5	5	2	1	5	1	1	5	1	2	1	45	16
[redacted]	1	1	4	1	1	5	2	1	2	1	5	5	4	5	1	2	3	44	18
[redacted]	3	2	3	3	1	1	5	5	1	2	5	1	1	5	1	2	3	44	18
[redacted]	1	1	4	1	3	5	1	1	1	1	5	5	2	5	1	4	2	43	20
[redacted]	2	1	3	1	2	5	5	5	2	1	5	1	1	3	1	1	3	42	21
[redacted]	2	1	3	1	2	5	1	3	2	1	5	5	1	5	1	2	1	41	22
[redacted]	3	1	3	1	1	5	5	1	4	1	1	1	3	5	1	2	3	41	22
[redacted]	2	1	2	1	1	5	3	2	2	1	5	1	3	5	1	3	3	41	22
[redacted]	1	1	3	1	2	5	1	3	1	1	5	1	3	5	1	3	4	41	22
[redacted]	1	1	1	1	1	5	5	2	1	1	5	1	2	5	1	2	1	36	26

DISTRICTS SCORECARD OF SELECTED INDICATORS, 2ND QUARTER 2017, ER.

DISTRICT	OPD per capita	Percentage of Teenage Pregnancies among ANC registrants	Family Planning Acceptor rate	Percentage skilled deliveries	Measles - Rubella 2 (MR-2) cov	Under 5 Malaria Case Fatality Rate	% Pregnant women tested HIV positive(PMTCT)	Penta 3 coverage	IPT	ANC	Authorisation Compltеness	Authorisation Timeliness	IDSR Weekly Timeliness	IDSR Monthly Compltеness	Non Polio AFP Rate	Data Entry Compltеness	Data Entry Timeliness	G.Total	Postion
[redacted]	5	3	1	5	4	5	5	5	1	5	3	5	1	4	5	5	5	67	1
[redacted]	5	3	2	5	5	5	5	5	5	1	3	5	3	1	3	5	5	66	2
[redacted]	5	2	1	5	2	5	4	2	2	5	4	5	5	4	5	5	5	66	3
[redacted]	2	1	3	1	5	5	4	5	5	2	4	5	5	4	5	1	5	62	4
[redacted]	5	2	4	4	5	5	2	5	2	2	3	5	1	3	4	5	5	62	4
[redacted]	5	1	1	5	1	5	5	3	2	3	4	5	5	3	5	1	5	59	6
[redacted]	1	3	1	1	5	5	1	5	2	1	4	5	4	4	5	5	5	57	7
[redacted]	1	1	3	1	5	5	5	5	2	1	4	5	1	2	5	5	5	56	8
[redacted]	4	2	1	5	3	5	1	4	2	4	3	5	1	3	3	5	5	56	8
[redacted]	3	2	2	3	2	5	5	4	1	2	3	5	3	4	4	1	5	54	10
[redacted]	2	1	3	1	3	3	5	5	2	1	3	5	1	5	3	5	5	53	11
[redacted]	3	1	3	2	2	5	5	3	3	1	4	5	1	4	4	1	5	52	12
[redacted]	2	1	5	1	5	5	1	5	2	1	4	5	5	3	4	1	1	51	13
[redacted]	5	2	3	5	1	5	3	1	3	1	2	5	1	4	4	1	5	51	13
[redacted]	1	1	5	1	2	1	5	2	3	1	3	5	1	4	5	5	5	50	15
[redacted]	1	1	1	1	1	5	5	1	3	1	4	5	1	4	5	5	5	49	16
[redacted]	2	2	1	1	2	3	5	5	4	1	4	5	1	2	5	1	5	49	16
[redacted]	2	2	3	2	1	5	5	3	3	1	4	5	2	4	5	1	1	49	16
[redacted]	1	1	3	1	2	5	5	2	2	1	4	5	1	4	4	1	5	47	19
[redacted]	3	1	1	2	1	5	5	1	3	1	3	5	1	4	4	1	5	46	20
[redacted]	1	1	3	1	2	5	1	3	1	1	4	5	1	2	4	5	5	45	21
[redacted]	3	1	1	2	1	5	1	3	4	1	3	5	1	3	4	1	5	44	22
[redacted]	2	2	1	1	1	5	1	2	3	1	2	5	1	3	3	5	5	43	23
[redacted]	2	1	4	1	1	5	3	2	1	1	4	5	1	3	3	1	5	43	23
[redacted]	1	1	1	1	1	5	1	4	1	1	4	5	2	2	3	1	5	39	25
[redacted]	1	1	1	1	2	5	1	2	1	1	3	4	1	4	4	1	5	38	26

DISTRICTS SCORECARD OF SELECTED INDICATORS, 3RD QUARTER 2017,ER.

District	OPD per capita	Percentage of Teenage Pregnancies among ANC registrants	Family Planning Acceptor rate	Percentage skilled deliveries	Measles - Rubella 2 (MR-2) cov	Under 5 Malaria Case Fatality Rate	% Pregnant women tested HIV positive (PMTCT)	Penta 3 coverage	IPT	ANC	Authorisation Complteness	Authorisation Timeliness	IDSR Weekly Timeliness	IDSR Monthly Complteness	Non Polio AFP Rate	Data Entry Complteness	Data Entry Timeliness	G.Total	Position
[redacted]	5	4	1	5	5	5	5	5	1	5	5	5	4	5	5	5	5	75	1
[redacted]	2	1	3	1	5	5	3	5	3	1	5	5	4	5	5	4	4	61	2
[redacted]	5	1	3	3	5	5	1	5	4	2	5	5	3	5	1	3	5	61	3
[redacted]	5	2	1	5	1	5	1	4	2	5	5	5	4	5	1	5	5	61	3
[redacted]	1	1	3	1	4	5	5	5	3	1	5	5	4	5	5	3	4	60	5
[redacted]	5	4	2	4	4	5	5	5	3	2	5	1	4	5	1	1	4	60	5
[redacted]	2	2	3	1	1	5	5	4	3	1	5	5	4	5	5	3	5	59	7
[redacted]	2	2	1	1	5	5	5	5	3	3	5	1	4	5	1	5	5	58	8
[redacted]	5	2	2	3	1	5	2	2	2	1	5	5	3	5	5	5	4	57	9
[redacted]	4	1	1	1	1	5	4	1	4	1	5	5	3	5	5	5	5	56	10
[redacted]	1	1	3	1	3	5	5	5	1	1	5	5	4	5	5	3	3	56	10
[redacted]	1	1	1	1	5	5	4	5	3	1	5	5	4	5	1	3	5	55	12
[redacted]	3	2	1	1	3	5	4	5	3	1	5	3	4	5	1	4	5	55	13
[redacted]	3	1	1	1	1	5	5	2	3	2	5	5	4	5	5	3	3	54	14
[redacted]	2	3	5	1	1	5	4	4	3	1	5	1	4	5	1	5	4	54	14
[redacted]	1	1	4	1	4	5	1	5	1	1	5	5	4	5	1	5	5	54	14
[redacted]	1	1	2	1	1	5	5	5	1	1	5	5	4	5	5	3	3	53	17
[redacted]	2	1	3	1	2	5	5	4	1	1	5	5	4	5	1	3	5	53	17
[redacted]	1	1	3	1	2	5	5	3	1	1	5	1	4	5	5	5	5	53	17
[redacted]	3	1	1	5	4	5	1	4	3	3	5	1	4	5	1	4	3	53	17
[redacted]	3	2	2	2	2	1	2	5	1	1	5	5	4	5	1	5	5	51	21
[redacted]	2	1	1	1	1	5	1	2	2	1	5	5	4	5	5	4	3	48	22
[redacted]	3	2	1	4	1	1	5	5	1	3	5	1	2	5	1	5	3	48	22
[redacted]	2	1	1	1	1	5	1	2	2	1	5	5	4	5	1	5	5	47	24
[redacted]	1	1	1	1	1	5	1	5	1	1	5	1	4	5	1	3	3	40	25

DISTRICTS SCORECARD OF SELECTED INDICATORS, 4TH QUARTER 2017,ER.

District	OPD per capita	Percentage of Teenage Pregnancies among ANC registrants	Family Planning Acceptor rate	Percentage skilled deliveries	Measles-Rubella2 (MR-2) cov	Under 5 Malaria Case Fatality Rate	% Pregnant women tested HIV positive(PMTCT)	Penta 3 coverage	IPT 3	ANC Coverage	Authorisation Complteness	Authorisation Timeliness	IDSR Weekly Timeliness	IDSR Monthly Compltepness	Non Polio AFP Rate	Data Entry Compltepness	Data Entry Timeliness	G Total	Postion
[redacted]	5	3	1	5	5	5	5	5	1	5	5	5	5	5	3	5	5	73	1
[redacted]	5	1	1	5	5	5	5	5	1	3	5	5	5	5	5	5	4	70	2
[redacted]	1	1	1	1	5	5	5	5	2	5	5	5	5	5	5	5	5	66	3
[redacted]	5	2	3	4	5	5	5	5	5	2	5	1	5	5	1	3	3	64	4
[redacted]	5	4	1	5	5	5	5	5	3	2	5	5	5	5	1	1	1	63	5
[redacted]	1	1	4	1	5	5	5	5	1	2	5	5	5	5	3	5	4	62	6
[redacted]	2	3	5	1	1	3	5	4	5	1	5	5	5	5	1	5	5	61	7
[redacted]	5	2	3	4	3	5	4	2	1	1	5	5	5	5	1	5	5	61	7
[redacted]	3	3	1	1	5	5	5	5	3	1	5	5	5	5	1	4	3	60	9
[redacted]	1	1	1	1	5	5	5	5	3	1	5	5	5	5	1	5	5	59	10
[redacted]	3	2	1	3	5	5	1	5	1	2	5	5	5	5	1	5	5	59	10
[redacted]	4	2	1	5	2	1	1	5	2	5	5	5	5	5	3	3	3	57	12
[redacted]	3	1	1	1	5	5	1	4	3	1	5	5	5	5	3	4	4	56	13
[redacted]	2	1	3	1	2	5	4	3	1	1	5	5	5	5	5	3	3	54	14
[redacted]	1	1	3	1	5	1	5	5	3	1	5	5	5	5	2	3	3	54	14
[redacted]	1	2	1	1	5	5	5	5	1	1	5	1	5	5	1	4	4	52	16
[redacted]	4	2	1	1	1	5	5	1	1	1	5	5	4	5	1	5	5	52	16
[redacted]	2	1	1	1	1	5	5	1	2	1	5	5	5	5	3	4	3	50	18
[redacted]	4	1	1	5	2	5	1	4	3	2	5	1	5	4	1	3	3	50	18
[redacted]	1	1	1	1	1	5	5	1	2	1	5	5	4	5	1	5	5	49	20
[redacted]	1	1	1	1	4	5	1	5	1	1	5	5	5	5	1	4	3	49	20
[redacted]	2	2	1	1	2	5	3	4	2	1	5	5	5	5	1	3	2	49	20
[redacted]	1	1	4	1	3	5	1	3	1	1	5	5	5	5	1	4	3	49	20
[redacted]	2	3	4	1	5	1	4	4	2	1	1	1	5	5	1	3	3	46	24
[redacted]	1	1	2	1	2	5	1	5	1	1	5	1	5	5	1	3	3	43	25
[redacted]	1	1	2	1	1	1	5	2	1	1	5	1	5	5	1	5	5	43	26

DISTRICT LEAGUE TABLE, JAN-DEC 2017, ER.

District	QTR 1	QTR 2	QTR 3	QTR 4	G. Total	Average	Postn.
[redacted]	65	67	75	73	280	70.00	1
	62	66	61	57	246	61.50	2
	57	66	60	63	246	61.50	2
	58	62	61	64	245	61.25	4
	58	62	58	66	244	61.00	5
	60	59	48	70	237	59.25	6
	52	51	61	62	226	56.50	7
	52	57	55	59	223	55.75	8
	47	51	57	61	216	54.00	9
	48	49	55	60	212	53.00	10
	56	44	54	56	210	52.50	11
	42	53	60	54	209	52.25	12
	45	49	54	61	209	52.25	12
	51	52	59	46	208	52.00	14
	44	54	51	59	208	52.00	14
	46	56	56	49	207	51.75	16
	46	56	53	50	205	51.25	17
	48	43	53	54	198	49.50	18
	43	50	54	49	196	49.00	19
	41	46	56	52	195	48.75	20
	44	49	47	49	189	47.25	21
	45	47	53	43	188	47.00	22
	41	45	53	43	182	45.50	23
	41	38	48	50	177	44.25	24
	41	43	39	49	172	43.00	25
	36	39	40	52	167	41.75	26
