# Supplementary material for: Managerial capacity among district health managers and its association with district performance: A comparative descriptive study of six districts in the Eastern Region of Ghana
Source: PLoS One. 2020 Jan 22;15(1):e0227974. doi: 10.1371/journal.pone.0227974 (PMC6975551; doi:10.1371/journal.pone.0227974)
Supplement: Attachment — Submitted filename: Response to reviewers, 14Oct.docx [file pone.0227974.s003.docx]

**Response to reviewers, PLOS ONE, PONE -D-19-20019**

The authors would like to sincerely thank the reviewers for their useful comments and suggestions, which enable us to improve the presentation of our study and the quality of our manuscript. Please find below our point-by-point responses to the concerns raised by the reviewers and how we addressed these concerns.

| **General comments** | **Authors’ responses and text changes (marked yellow in the manuscript)** |
| --- | --- |
| Clarify the concept of “District Health Management” in introduction and also modify it in method and title to avoid misunderstandings. In some case District Health Management means a structure of management and in your case it means the head managers of this structure not the management as a mechanism and structure. | This is a valuable comment. Consequently, we have modified the title, abstract, introduction and method section to reflect that district health management in the current study relates to the managerial capacity of the district health managers and their assessment of the system they work in.  *Please see highlighted text line 4, 29, 88, 175* |
| Clarify whether you consider the role and capabilities of district health managers as district health management role and capabilities in discussion and limitation section | We are not certain what is meant by this comment; however we understand it as being closely related to the previous one. We have clarified in the discussion section that the study refers to management competencies among district health managers. We hope this responds to the reviewers comment. If not, please provide further clarification. Thank you  *Please see highlighted text line 352, 376* |
| You can find new references instead of old one. I recommend you discuss and compare your findings with the result of a series of paper we published in relation to district health management as a similar setting in a developing country. In this project we investigate the district health management environment in a developing country (Managerial barriers and challenges in Iran public health system: East Azerbaijan health managers' perspective. J Pak Med Assoc) after that develop management training program (Developing management capacity building package to district health manager in northwest of iran: a sequential mixed method study. J Pak Med Assoc), then design a management performance framework (A framework to assess management performance in district health systems: a qualitative and quantitative case study in Iran. Cadernos de saude publica) and finally implement and evaluate training programme (Evaluation of the district health management fellowship training programme: a case study in Iran. BMJ open). | We thank the reviewer for drawing this literature relating to Azerbaijan and Iran to our attention. Findings from these studies have been incorporated in the discussion section of the manuscript.  *Please see highlighted text lines 289, 298, 309, 311, 315, 324, 330, 347* |
| Clarify the table topics, such a “high”, “low”, “A”, “B”,… without any directive explanation. | Based on the comments from reviewer 1 and 4, the analyses have been modified; instead of stratifying the analysis by low, mid and high performing districts we use the districts’ performance scores directly. Consequently the table topics have been modified. “High”, “low”, “A” “B” does not appear anymore in the revised version of the manuscript.  *Please see table 3, 4 and 5.* |
| Provide self-administered questionnaire, study protocol, informed consent as appendix. | The listed items have been added as appendix to our submission |
| Describe the self-administered questionnaire validation process in detail (how many expert, Validation methods, Analysis, …). | The validation process has been described in more detail, and now outlines the questionnaire development process, including literature review, qualitative interviews, the number of experts and the cognitive validation methods.  *Please see highlighted text line 125-138, 378-380.* |
| Describe cutoff point for Cronbach’s alpha coefficient and the result of your study. | The Cronbach’s alpha coefficient was applied to test the internal consistency of the sum scales relating to specific health system management domains to ensure a reliability score of >0.7, based on statistical literature which have been referenced.  *Please see highlighted text line 165-166*  Cronbach alpha coefficients were calculated for the scales that had not been validated in previous studies, and the results are listed below figure 1 and table 5.  *Please see Figure 1 (line 247-248), Table 5 (line 270-275)* |
| Provide detailed information in relation to District Health Information Management System (DHIMS2) analysis method and process as appendix. | An appendix describing the analysis method for the league table of district performance has been attached as an appendix to the manuscript. |
| Consider confounding factor in your analysis and provide more detail in relation to controlling their in Statistical analysis section. | With the total sample size of 59 respondents, it is not practical to control for confounding through stratification or modelling. We have clarified that the analyses in current study are unadjusted.  *Please see highlighted text, line 172-178*  However, Table 3 and 4 demonstrates that there are no obvious patterns in the distribution of the most plausible potential confounding factors across districts. This has been elaborated in the discussion.  *Please see highlighted text 403-407*  Confounders, which were not adjusted for in current study, have been elaborated in the discussion.  *Please see highlighted text, line 357ff* |
| **Reviewer #1** |  |
| My primary concern relates to the generalizability of the study beyond those surveyed. The study was conducted in one region in the country. Further, in that region only 6 out 26 districts were assessed. | We thank the reviewer for their time and inputs. We recognize the concern regarding generalizability of the study findings and conclusions given that 6 districts in Ghana were included. After discussion with the Eastern Regional Director of Health Services, we confidently argue that the selected study districts reflect general patterns in the region. However, we agree with the need add additional information about the selection of study districts and discuss generalizability further in the discussion. We have consequently revised our manuscript.  *Please see highlighted text line 98-101, line 409-411* |
| Why was there a misalignment between the findings based on perceived management capacity and the objective measures of management competencies? Perhaps,  (a) an association between self-reported capacity was observed primarily because the majority of the district managers in the low performing districts reported little prior management experience or training (as reported). Thus, these managers, because of the lack of training or experience viewed themselves as lacking in management skills, even though they may not have differed from other managers. (b) There really was not enough variation in the performance measure to warrant the classification of three distinct groups (low medium, high). The cut-off points for these felt a bit arbitrary. (c) Also it is possible that the lack of statistical significance across several of the indicators assessed may be due to a small sample size. I would love to see the authors discuss these above-mentioned issues a bit more. | We thank the reviewer for their thoughtful inputs.  First, we want to address the second point of the reviewer, namely the classification of district groups. We have redone the analyses without reliance on the arbitrary groupings, using instead the districts’ performance scores directly. However, our findings remain the same, and we thus agree that the manuscript would benefit from a more detailed discussion of the discrepancy between the overall rating of management capacity and the domain specific ratings, as well as the discrepancy between the domain specific ratings and the objective measure of district performance.  Guided by the comments of the reviewer, we have discussed the potential impact of the following issues 1) few respondents across several of the indicators, 2) exposure to domain specific management training 3) how the overall assessment of management capacity and district performance may be impacted by dynamics within the District Health Management Team.  *Please see highlighted text line 333-344.* |
| Abstract: Aim: May be missing "competencies" after "management" | Thank you for bringing this to our attention. We have revised the manuscript accordingly.  *Please see highlighted text, line 29.* |
| Figure 1: Indicates (lines 234-237) that low performing districts have lower scores in time planning than mid-performing. The figure as shown indicates little difference between the two groups. Suggest that the authors exclude time planning from the list. | Thank you for bringing this to our attention. We have revised the manuscript accordingly by excluding time planning from the list.    *Please see highlighted text, line 239.* |
| **Reviewer #2** |  |
| The downside is that this manuscript is very similar to the one by Fetene N et al (note the misspelling on line 74) who studied district-level health management and health system performance in Ethiopia. | As the reviewer points out, the study of Fetene et al took place in Ethiopia. The health systems in Ethiopia and Ghana have vast differences, and we thus argue that our study adds value by exploring district health management characteristics and analyzing the association between management capacity among district health managers and performance in a Ghanaian context. Moreover, the two studies rely on and use different approaches to measure management capacity among district health managers.  We also thank the reviewer for drawing attention to the mispelling, which has been corrected *(line 74)* |
| The outcome variable, district performance, was not part of the questionnaire, rather it was extracted from aggregated data collected annually, so any association between the dependent and independent variables is questionable. | As stated in the manuscript, causality between the independent variables and the outcome variable cannot be established (*line 390ff*). However, we argue that our aim to explore the association between managerial capacity among district health managers and district performance as measured by the Ghana League Table of District Performance is relevant, as the district performance score currently is applied by the national and regional level to assess how well District Health Management Teams perform  *No changes have been made in the manuscript based on this comment* |
| Also, the authors did not investigate district health management implementers (such as doctors, nurses) and beneficiaries (such as patients), so the study was subjective and has no promotional value | Managerial competencies in our study were based on self-assessment, using the same approach as other studies. The limitations, including subjectivity, have been discussed (*line 368ff*). However, in spite of the limitations, we argue that the findings are of value and practical relevance for the improvement of district health management in Ghana. Firstly, this study highlights critical shortcomings in terms of management training, management support systems, district health managers’ motivation etc. Secondly, the self-assessed management competencies can be applied as an initial indicator of management capacity among district health managers in Ghana.  *No changes have been made in the manuscript based on this comment* |
| It is unclear how the authors actually assessed the outcome variables (high, medium, and low levels). In the methods section, the measurement standard cannot be found. What were the cut-points for high, medium and low and why were they chosen? In other words, the authors need to make a reasonable explanation for classifying the districts with scores of 61.5 and 56.5 into the high performance group, 52.3 and 52.0 into the middle performance group, and 47.0 and 43.0 into the low performance group. | This study is an integral part of the larger project PERFORM2Scale. The PERFORM2Scale districts were among other factors selected based on their different level of performance (poor, moderate and good) (line 97-100) ranked by the Regional Health Administration. We grouped the districts similarly to how they had been grouped in PERFORM2Scale terms, however recognize the reviewers’ point. Thus, we have redone the analyses without reliance on the arbitrary PERFORM2Scale groupings, using instead the districts’ performance ranking scores approved by Ghana Health Service. Our findings remain the same.  *Please see highlighted changes in table 3, 4 and 5.* |
| The authors need to explain whether this is a random cluster sampling study or a convenience cluster sampling study. There is also no calculation of the sample size and exclusion criteria for the study in this manuscript. | The study districts were sampled through their involvement in the PERFORM2Scale project in which districts were selected by researchers from the School of Public Health, University of Ghana and the Eastern Regional Health Administration.  All eligible managers within the selected PERFORM2Scale districts (*line 107-109*) were contacted and over 95% responded. Hence there was no sample calculation to perform.  We have added additional information on how the districts were selected for the PERFORM2Scale.  *Please see highlighted text line 98-101.* |
| **Reviewer #3** |  |
| The study is important in terms of management and health system performance in Ghana. The statistical analysis reflects the answers to the tasks set in the study. The author highlights the main problems but also reflects some limitations of the study which did not allow to fully elucidate the proposed goal. | We thank the reviewer for taking the time to review our paper |
| **Reviewer #4** |  |
| I will suggest you revisit your statistical analysis, everything is wrong about it. Why did you choose non-parametric test and not ANOVA parametric test? Your data is two level hierarchical in nature where the 59 managers were nested in 6 district, you will need to account for this clustering in your analysis. SO therefore, I was thinking you should have applied some kind of multivariable logistic model while adjusting for clustering in 6 district. Also, considering you categorized your outcome i.e. dependent variable into three level, you will need to applied multinomial logistic regression to this data. | Thank you for your comments, and taking the time to review our paper.  We used non-parametric tests for the analysis of our quantitative variables because most of them come from the Likert scale and show strong departures from normality.  While it is true that our 59 subjects are grouped in 6 districts, the main analysis associates a subject-level variable (individual managerial competencies) with a district-level variable (district performance score). Therefore, typical adjustments for intrinsic cluster effects (for example via mixed models) cannot be applied. By the same token, there is little added value in regression modelling of this association compared to the analysis of between-district differences which we perform.  *Please see highlighted text changes line 177-178* |
| That Table three is not right at all, you can not be applying kruskal-wallis test (for testing continuous variables) to categorical variables like sex, educational background, educational qualification, year in current position etc No you cant, this is slap on statistic face. | The use of the Kruskal-Wallis test for the analysis of the distribution of categorical characteristics across districts is indeed incorrect, and we thank the reviewer for drawing our attention to this.  Instead, we have instead applied the Fisher’s exact test which is appropriate for our data given the small number of counts in many cells.  *Please see changes in table 3* |
| How on earth did you apply nptrend to ordered data when its not a time-series in nature? Why? | The trend test is applicable to any data arranged in ordered groups, and the order needs not be temporal. Put simply, not every trend is a time trend. In our case, it demonstrates that the (self-reported) managerial competence grows with the performance score of the district in a statistically significant way.  We stand by the choice and application of this method.  The reference for this test has been inserted in the manuscript (Cuzick 1985, A Wilcoxon-type test for trend).  *Please see highlighted text line 177.* |
| Your best bet: Assuming you dont know how to do all I described above, please use ordinary CHI-SQUARE TEST, apply it to Table 3. Considering your sample size was low (n=59), please categorize, the performance outcome into two levels i.e. High vs mid/Low (combine Mid/Low together) then apply Chi-square test to EACH of the categorical variable. Then test Age (continuous variable using ANOVA) or you can categorize Age into 2 or 3 levels and then apply Chi-square test. Then this will at least suffice. But it will be better to apply Multivariable logistic regression/ Mixed effect logistic model to this data. | Thank you for your guidance. Nevertheless, as stated in the comment above regarding table 3, a Fisher’s exact test has been applied due to the number of cell counts.  Moreover, we opted not to group the districts into two categories as suggested by the reviewer. This would prevent us from taking full advantage of the dataset.  As explained above, the multivariable regression analysis is not optimal for our dataset.  *Please see changes in table 3* |
