# Supplementary material for: Managerial capacity among district health managers and its association with district performance: A comparative descriptive study of six districts in the Eastern Region of Ghana
Source: PLoS One. 2020 Jan 22;15(1):e0227974. doi: 10.1371/journal.pone.0227974 (PMC6975551; doi:10.1371/journal.pone.0227974)
Supplement: Attachment — Submitted filename: PLOS ONE, Letter to Editor, 10Dec19.docx [file pone.0227974.s004.docx]

Basel, 10 December 2019

PLOS ONE

**Re: PONE-D-19-20019R1**

**District Health Management and its association with District Performance: A comparative descriptive study of six districts in the Eastern Region of Ghana**

Dear Kamal Gholipour,

Thank you for your email dated the 6^th^ of December 2019 including the assessment of the revised manuscript entitled “*District Health Management and its association with District Performance: A comparative descriptive study of six districts in the Eastern Region of Ghana*”.

In your response, you kindly invited us to conduct minor revisions, including 1) checking that all statistic values were included in the tables and 2) formatting the reference list. Please find attached the revised manuscript, in which these two comments have been addressed.

We remain at your disposal to clarify any further questions, and look forward to your final decision. Thank you very much for your consideration.

Yours sincerely,

Anne Christine Heerdegen, corresponding author
